# Two-stage learning-based prediction of bronchopulmonary dysplasia in very low birth weight infants: a nationwide cohort study

**DOI:** 10.3389/fped.2023.1155921

**Published:** 2023-06-13

**Authors:** Jae Kyoon Hwang, Dae Hyun Kim, Jae Yoon Na, Joonhyuk Son, Yoon Ju Oh, Donggoo Jung, Chang-Ryul Kim, Tae Hyun Kim, Hyun-Kyung Park

**Affiliations:** ^1^ Department of Pediatrics, Hanyang University College of Medicine, Seoul, Republic of Korea; ^2^ Department of Artificial Intelligence, Hanyang University, Seoul, Republic of Korea; ^3^ Department of Pediatric Surgery, Hanyang University College of Medicine, Seoul, Republic of Korea; ^4^ Department of Computer Science, Hanyang University, Seoul, Republic of Korea

**Keywords:** machine learning—ML, bronchopulmonary dysplasia (BPD), prediction, very low birth weight infants (VLBWI), nationwide cohort

## Abstract

**Introduction:**

The aim of this study is to develop an enhanced machine learning-based prediction models for bronchopulmonary dysplasia (BPD) and its severity through a two-stage approach integrated with the duration of respiratory support (RSd) using prenatal and early postnatal variables from a nationwide very low birth weight (VLBW) infant cohort.

**Methods:**

We included 16,384 VLBW infants admitted to the neonatal intensive care unit (*N*ICU) of the Korean Neonatal Network (KNN), a nationwide VLBW infant registry (2013–2020). Overall, 45 prenatal and early perinatal clinical variables were selected. A multilayer perceptron (MLP)-based network analysis, which was recently introduced to predict diseases in preterm infants, was used for modeling and a stepwise approach. Additionally, we applied a complementary MLP network and established new BPD prediction models (PMbpd). The performances of the models were compared using the area under the receiver operating characteristic curve (AUROC) values. The Shapley method was used to determine the contribution of each variable.

**Results:**

We included 11,177 VLBW infants (3,724 without BPD (BPD 0), 3,383 with mild BPD (BPD 1), 1,375 with moderate BPD (BPD 2), and 2,695 with severe BPD (BPD 3) cases). Compared to conventional machine learning (ML) models, our PMbpd and two-stage PMbpd with RSd (TS-PMbpd) model outperformed both binary (0 vs. 1,2,3; 0,1 vs. 2,3; 0,1,2 vs. 3) and each severity (0 vs. 1 vs. 2 vs. 3) prediction (AUROC = 0.895 and 0.897, 0.824 and 0.825, 0.828 and 0.823, 0.783, and 0.786, respectively). GA, birth weight, and patent ductus arteriosus (PDA) treatment were significant variables for the occurrence of BPD. Birth weight, low blood pressure, and intraventricular hemorrhage were significant for BPD ≥2, birth weight, low blood pressure, and PDA ligation for BPD ≥3. GA, birth weight, and pulmonary hypertension were the principal variables that predicted BPD severity in VLBW infants.

**Conclusions:**

We developed a new two-stage ML model reflecting crucial BPD indicators (RSd) and found significant clinical variables for the early prediction of BPD and its severity with high predictive accuracy. Our model can be used as an adjunctive predictive model in the practical NICU field.

## 1. Introduction

Despite the advances in respiratory care, the incidence of bronchopulmonary dysplasia (BPD) is increasing with the increase in the survival rate of extremely premature infants born at immature stages of lung development (1–3). As survivors with BPD undergo longer hospitalization with an increase in readmission after discharge and a high risk of poor pulmonary and neurodevelopmental outcomes (4, 5), the early identification of the risk of developing BPD is imperative for preventive interventions.

The commonly used National Institute of Child Health and Human Development (NICHD) criteria cannot determine the BPD severity until the postmenstrual age of 36 weeks (6). Thus, several models for predicting BPD have been established using birth weight (BW), gestational age (GA), sex, patent ductus arteriosus (PDA), sepsis, artificial ventilation, etc. to estimate the probability of BPD occurrence and optimize BPD treatment strategies (3, 7, 8). The majority of the existing models use traditional statistics (multiple logistic regression) or commercial machine learning (ML) methods, pay little attention to BPD severity, and are based on small sample populations (7–9). In addition, the fact that deceased patients were excluded from prediction model development was pointed out as a limitation.

Recently, artificial intelligence (AI) models have become promising prediction tools and have been used in several clinical applications (10–12); however, the use of machine learning (ML) algorithms is still limited in the field of neonatology. In previous studies, we showed efficient performance of the PDA prediction tasks of machine learning models (11), and then developed our new artificial neural networks (ANNs) for predicting intestinal perforation using very low birth weight (VLBW) infant data from a nationwide cohort (12). Therefore, we intend to apply our multilayer perceptron (MLP)-based experience and enhance its performance using a two-stage approach with one of the imperative variables for BPD occurrence to maximize clinical feasibility.

This study aimed to develop new ML models for the early prediction of BPD and its severity (BPD prediction model, PMbpd) using prenatal and early perinatal clinical variables obtained from a nationwide VLBW infant cohort and to compare their performance with that of classic ML models. Furthermore, we optimized the prediction model by building a two-stage approach through the first step of prediction using the duration of respiratory support (RSd), which is closely related to the BPD risk (13–15).

## 2. Materials and methods

### 2.1. Patients and data collection

This study investigated the database provided by the Korean Neonatal Network (KNN), a nationwide prospective registry of VLBW infants. The KNN consists of 77 tertiary hospitals, covering approximately 75%–80% of VLBW infants born in South Korea. It has enrolled VLBW infants, preterm infants born with birth weights of less than 1,500 g, or those transferred within 28 days after birth to registered neonatal intensive care units (NICUs) since 2013. This study was approved by the Hanyang University Institutional Review Board (IRB No. 2013-06-025-043). The inclusion criteria for this analysis was all VLBWIs from KNN data. Exclusion criteria are those that are more than 32 weeks old, or infants with major congenital anomalies, or has unclear data. It also excluded cases of death prior to 36 weeks, but deaths from BPD were included in the severe BPD.

### 2.2. Clinical variables and definition

In the KNN database, each chief neonatologist of the participating NICUs provided information regarding the data. The KNN network collects demographic, environmental, and clinical variables of VLBW infants from the prenatal period to 36 months of corrected age. The neonatologists reviewed the published literature and selected the potential variables. Overall, 45 variables were selected and modified from the database and classified as continuous or discrete (categorical or ordinal).

We used the BPD definition based on the 2001 NICHD criteria (6). No BPD (BPD 0) was defined as <28 days of supplemental oxygen intake. Mild BPD (BPD 1) included infants who received oxygen or respiratory support for >28 days but were on room air at 36 weeks postmenstrual age (PMA). Infants with moderate BPD (BPD 2) required supplemental oxygen and a <30% fraction of inspired oxygen concentration at 36 weeks PMA. Finally, severe BPD (BPD 3) was classified as the use of >30% oxygen or positive pressure at 36 weeks’ PMA or death before 36 weeks' PMA from BPD.

RSd was defined as the duration of invasive ventilation in days. PDA treatment was defined as PDA with any treatment, and low blood pressure (BP) was defined as hypotension with medication within the first week of age. Sepsis was defined as a confirmed infection within the first week of life. Intraventricular hemorrhage (IVH) was defined using Papile's criteria and cranial ultrasonography (16). The majority of IVHs develop within 3 days, and PDA also affects the early postnatal period; therefore, these factors were included. Pulmonary hypertension (PHT) was defined as whether PHT was suspected or confirmed by echocardiography or clinically and was treated with medication within 1 week of age. The complete list of the 45 variables used in the analysis is shown in the Supplementary (Table S1) and was selected from the database based on the existing literature using the KNN database (12, 17).

### 2.3. Statistical analysis

The chi-square test and one-way analysis of variance (ANOVA) were used to compare the demographic and clinical characteristics among the four BPD severity levels. A *P*-value <0.05 were considered significant for all statistical analyses. Statistical analyses were performed using SPSS, version 26.0 (IBM, Armonk, New York, USA).

### 2.4. Machine learning prediction model development

#### 2.4.1. Classic machine learning algorithms

Several classic ML algorithms can handle disease prediction problems. Therefore, we chose several algorithms to confirm the diagnostic performance of classic ML algorithms for comparison with our proposed models. Predictions were conducted using the linear Support Vector Machine (SVM), radial SVM, logistic regression, k-Nearest Neighbor (k-NN), decision tree, Extreme Gradient Boost (XGBOOST), and Light Gradient Boost Machine (GBM) methods. We used the *xgboost* library for XGBOOST and the *lightgbm* library for Light GBM, and the remaining algorithms were obtained from the *Scikit-Learn library*.

#### 2.4.2. Data preprocessing

The data preprocessing step before training is essential for improved training and performance in data-limited situations. First, among the 416 variables that can be obtained from KNN, we excluded variables in which more than half of the missing value. Forty-five variables were determined by selecting and processing prenatal and early postnatal variables related to BPD. To fill in the missing values, we divided the variables into three types: continuous, nominal, and ordinal. The remaining missing values were filled with the means for the continuous type of variables and the modes (nominal or ordinal variables). Before training, with min-max normalization, we scaled the data between 0 and 1. Finally, the preprocessed data was divided into 0.9 and 0.1 ratios for training and validation data, and additionally, to prevent data bias, the dividing process was conducted class-wise.

#### 2.4.3. Training

With respect to training in the traditional and proposed approaches, the same preprocessed data were used for fairness. Traditional models were trained and evaluated using the *Scikit-Learn library,* and training was performed using default hyperparameter settings. When training MLP models, it is necessary to set hyperparameters, optimizers, and loss functions. Therefore, the proposed models were trained with the Adam optimizer (18), with a batch size of 128, a learning rate of 1e−3, and a dropout rate of 0.2. In addition, we used the mean squared error (MSE) loss for training instead of cross-entropy loss or binary cross-entropy loss, as setting the MSE loss for the objective function showed better performance in experiments. Although entropy-type losses are commonly used in training classification models, MSE losses are occasionally more effective (19). The training was conducted until the loss value of the evaluation did not decrease 10 times in a row, instead of fixing the training epoch. The parameters of the models were updated by backpropagating the MSE loss for PMbpd and TS-PMbpd. To aid the optimization process, we added dropout (20) and batch normalization (21) at each layer, except for the last one. All the settings aimed to improve the area under the receiver operating characteristic curve (AUROC) values, and to clarify each case's results, we added AUROC values and precision, recall, and f1-score. Our MLP models were implemented using the *PyTorch* library, and evaluations were performed using *the Scikit-Learn library*.

#### 2.4.4. Prediction model development (PMbpd, Ts-PMbpd)

**Figure 1:**
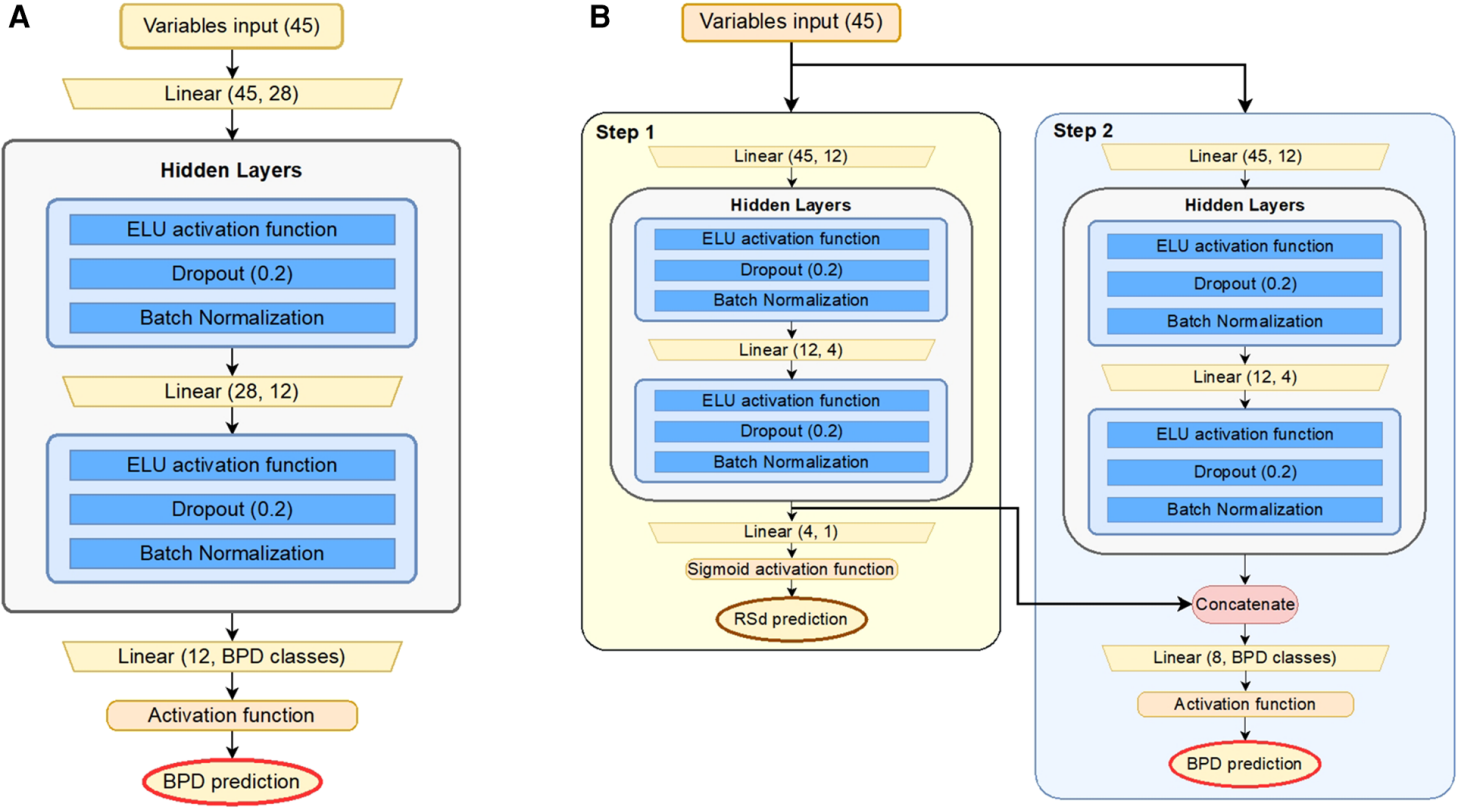
Structure of models: (**A**) pMbpd is a baseline neural network based on the conventional MLP architecture. (**B**) TS-PMbpd is composed of two different MLPs. In Step 1, one MLP predicts RSd, and in Step 2, the other MLP predicts BPD. The feature vectors from the second layer of the network for RSd are concatenated with the second layer of the MLP for BPD. The activation function of the last layer is sigmoid function for binary classification and softmax function for multi-classification. MLP, multilayer perceptron; BPD, bronchopulmonary dysplasia.

Traditional methods exist for accurate forecasts of BPD; however, improvements are required for accurate forecasts. In this study, we designed an ANN model for precise diagnoses, expecting neural networks to analyze extensive data on BPD more accurately. Because diagnosing BPD is a binary- or multi-classification problem that predicts the occurrence of BPD and types of BPD in infants from a given set of 45 variables, we started modeling from a simple MLP architecture that is widely used as a classifier. PMbpd is a one-dimensional input ANN model with a hidden layer (Figure 1A). The number of hidden layers was experimentally selected, and it exhibited high accuracy and stability during training. Our simple MLP model (PMbpd) showed excellent performance compared with traditional algorithms; however, we further developed PMbpd. The developed model is a two-stage model (TS-PMbpd) that uses information from the RSd to predict BPD severity. The TS-PMbpd consists of two MLP models and can be divided into two steps. In the first step, an MLP model predicted the RSd. In the next step, the final model uses input variables and a feature vector from the MLP model that predicts RSd by concatenation (12) at the hidden layer to forecast the BPD severity, as shown in Figure 1B. Because the feature vector from the first step MLP model contains information about RSd, which is a disease relevant to BPD, it helps to predict BPD severity. This could be confirmed by the improvement in performance for the BPD multi-classification problem, which is a problem with a small number of cases.

#### 2.4.5. Shapley additive exPlanation (SHAP)

The Shapley value was calculated to determine the input variables that significantly affected the judgment of the model (22). Although there are other approaches (e.g., permutation feature importance and coefficients as feature importance) to determine the contribution of variables to the predictive model, it is often difficult to apply them to ANNs for interpretability. The Shapley value is an approach based on cooperative game theory that can check the degree of positive or negative impact on all variables. To obtain convenient computations and explainable results, a calculation method called SHAP was used (23).

## 3. Results

### 3.1. Study population and data selection

**Figure 2:**
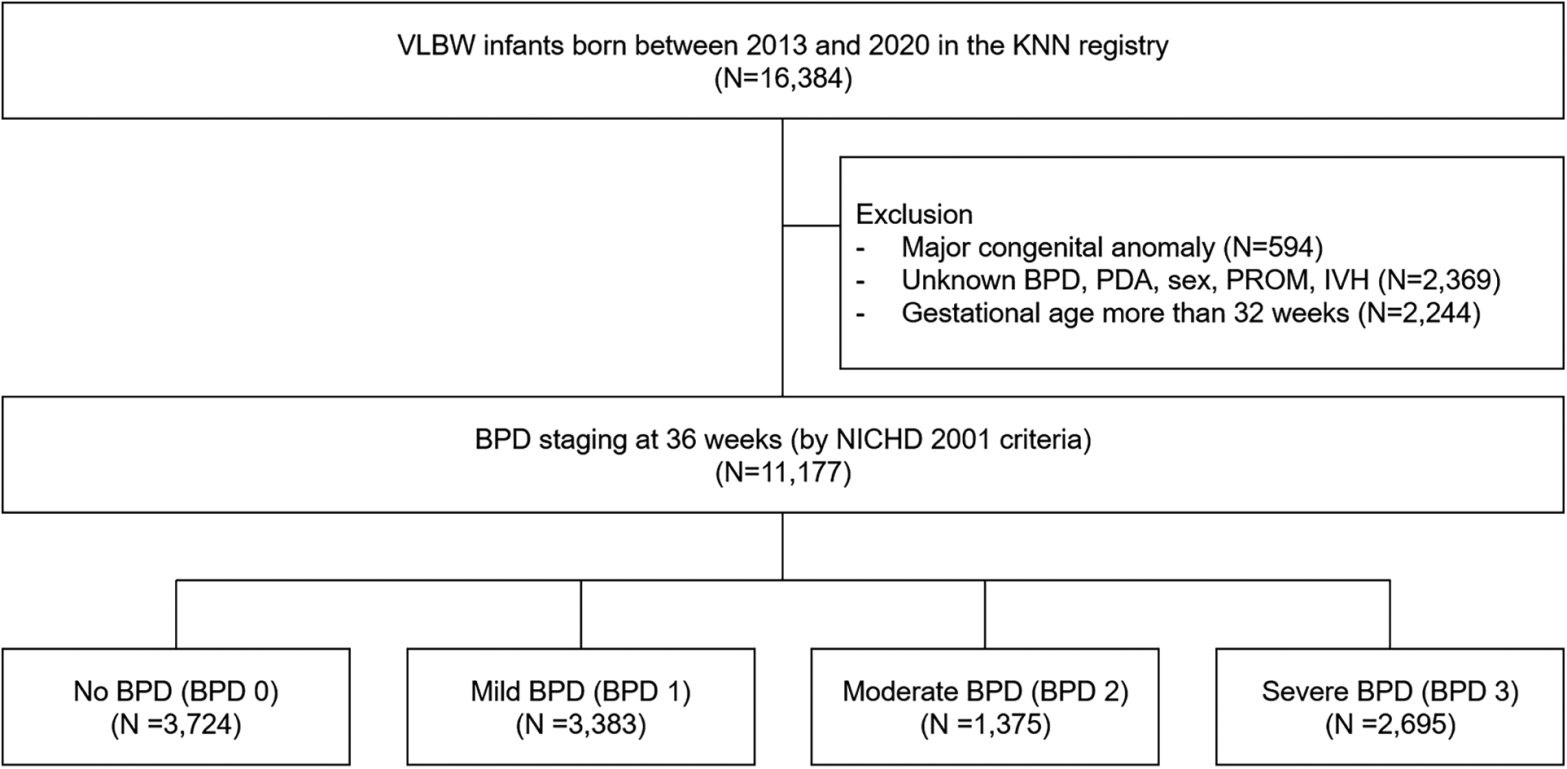
Flow diagram of the study population. KNN, Korean Neonatal Network; BPD, bronchopulmonary dysplasia; PDA, patent ductus arteriosus; PROM, premature rupture of membranes; IVH, intraventricular hemorrhage; NICHD, National Institute of Child Health and Human Development.

The study flowchart is shown in Figure 2. In total, 16,384 VLBW infants were enrolled in this prospective cohort. We excluded infants who were diagnosed with major congenital anomalies (*N* = 594), cases whose presence of BPD, PDA, sex, premature rupture of membrane, IVH was not specified (*N* = 2,369), and gestational age >32 weeks (*N* = 2,244). Patients who died before being diagnosed with BPD were excluded, but those who died due to BPD were included in BPD 3. The baseline demographic characteristics of the subjects are summarized in Table 1. All variables except maternal overt diabetes mellitus, maternal chronic hypertension and chorioamnionitis showed significant differences among the subgroups (*P *< 0.001). All the 45 variables stated in Supplementary (Table S2).

**Table 1:** Demographic and clinical characteristics of the study participants.

Variables	BPD 0 (*n* = 3,724)	BPD 1 (*n* = 3,383)	BPD 2 (*n* = 1,375)	BPD 3 (*n* = 2,695)	*P*-value
Sex (male)	1,718 (46.1)	1,673 (49.5)	743 (54.0)	1,465 (54.4)	<0.001
GA, weeks	30.04 ± 1.17	27.90 ± 1.65	27.61 ± 1.99	27.00 ± 2.12	<0.001
AS1	5.57 ± 1.79	4.53 ± 1.87	4.06 ± 1.83	3.95 ± 1.81	<0.001
AS5	7.61 ± 1.37	6.80 ± 1.66	6.37 ± 1.76	6.29 ± 1.79	<0.001
BW, gram	1,259.19 ± 183.51	1,064.39 ± 224.15	1,010.93 ± 245.57	885.79 ± 250.51	<0.001
BW_z	−0.01 ± 0.01	0.01 ± 0.54	0.06 ± 1.19	0.15 ± 1.84	<0.001
**DM**					
O_DM	48 (1.3)	48 (1.4)	21 (1.5)	44 (1.6)	0.709
A_DM	449 (12.1)	394 (11.6)	153 (11.1)	219 (8.1)	<0.001
**HTN**					
C_HTN	92 (2.5)	79 (2.3)	36 (2.6)	66 (2.4)	0.95
A_HTN	899 (24.1)	526 (15.5)	241 (17.5)	539 (20.0)	<0.001
CA	917 (24.6)	1,139 (33.7)	500 (36.4)	997 (37.0)	0.05
pH1h	7.27 ± 0.09	7.27 ± 0.10	7.26 ± 0.10	7.26 ± 0.11	<0.001
BE1h	−4.73 ± 3.16	−5.15 ± 3.38	−5.25 ± 3.47	−5.51 ± 3.62	<0.001
RDS	2,576 (69.2)	3,121 (92.3)	1,282 (93.2)	2,565 (95.2)	<0.001
SFT	2,543 (68.3)	3,161 (93.4)	1,291 (93.9)	2,575 (95.5)	<0.001
SFTnu	0.77 ± 0.61	1.20 ± 0.63	1.31 ± 0.79	1.38 ± 0.76	<0.001
PDATx	735 (19.7)	1,493 (44.1)	686 (49.9)	1,626 (60.3)	<0.001
PDALg	53 (1.4)	325 (9.6)	231 (16.8)	778 (28.9)	<0.001
PHT	21 (0.6)	78 (2.3)	76 (5.5)	453 (16.8)	<0.001
lowBP	195 (5.2)	652 (19.3)	412 (30.0)	1,237 (45.9)	<0.001

Values are expressed as numbers (%) or means (standard deviations). BPD, bronchopulmonary dysplasia; BPD 0, no BPD; BPD 1, mild BPD; BPD 2, moderate BPD; BPD 3, severe BPD; AS1, 1-minute Apgar score; AS5, 5-minute Apgar score; BW, birth weight; BW_z, birth weight z-score; O_DM, maternal overt diabetes mellitus; A_DM, maternal all types of diabetes mellitus; C_HTN, maternal chronic hypertension; A_HTN, maternal all types of hypertension; CA, histologic chorioamnionitis; pH1h, hydrogen ion concentration in the blood within 1 h after birth; BE1h, Base excess within 1 h after birth.

RDS, respiratory distress syndrome; SFT, need for surfactant; SFTnu, Number of administered surfactants; PDATx, patent ductus arteriosus with any treatment; PDALg, Patent ductus arteriosus with surgical closure; PHT, pulmonary hypertension with treatment within 1 week of age; low BP, hypotension with medication within 1 week of age.

### 3.2. Prediction performance between new PMbpd and classic ML models

**Figure 3:**
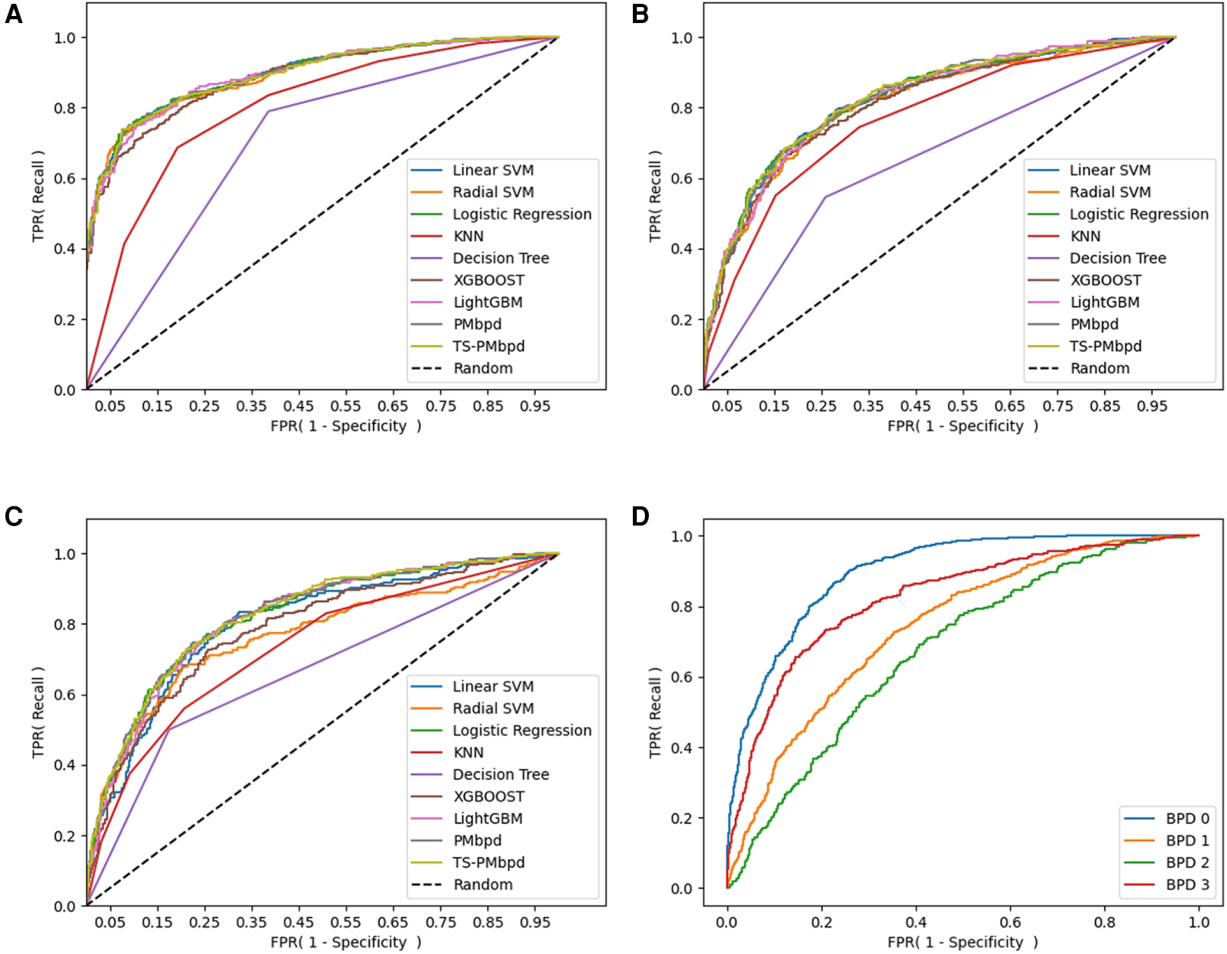
ROC curve of BPD prediction ML models: (**A**) ROC curve of BPD prediction ML models in binary classification (BPD 0 vs. BPD 1,2,3). (**B**) ROC curve of BPD prediction ML models in binary classification (BPD 0,1 vs. BPD 2,3). (**C**) ROC curve of BPD prediction ML models in binary classification (BPD 0,1,2 vs. BPD 3). (**D**) ROC curve of the TS-PMbpd model in multi-classification (BPD 0 vs. BPD 1 vs. BPD 2 vs. BPD 3). BPD, bronchopulmonary dysplasia; BPD 0, no BPD; BPD 1, mild BPD; BPD 2, moderate BPD; BPD 3, severe BPD.

The performance evaluation of the newly proposed BPD prediction model (PMbpd, TS-PMbpd) and other ML models applied in our study is summarized in Table 2. TS-PMbpd demonstrated outperformance about AUROC value and accuracy in the analysis of the diagnosis of BPD (0.8966, 0.8199, respectively). PMbpd demonstrated outperformance about F1-score and accuracy, and TS-PMbpd about AUROC value in the analysis of the diagnosis of BPD ≥2 (0.7754, 0.7764, 0.8253 respectively). PMbpd demonstrated outperformance about F1-score and AUROC values in the analysis of the presence of BPD 3 (0.7793, 0.8277, respectively). TS-PMbpd demonstrated outperformance about AUROC value and accuracy in the analysis of the diagnosis of each BPD severity (0.7855, 0.5912, respectively). Detailed performance results, including prediction, recall, F1-score, and accuracy, are summarized in Table 2, and the receiver operating characteristic (ROC) curves are shown in Figure 3.

**Table 2:** Comparisons of the performance in BPD and severity prediction.

	BPD 0 vs. BPD 1,2,3					BPD 0,1 vs. BPD 2,3					BPD 0,1,2 vs. BPD 3					BPD 0 vs. BPD 1 vs. BPD 2 vs. BPD 3				
	Precision	Recall	F1-score	AUROC	Accuracy	Precision	Recall	F1-score	AUROC	Accuracy	Precision	Recall	F1-score	AUROC	Accuracy	Precision	Recall	F1-score	AUROC	Accuracy
Linear SVM	0.8507	0.8632	0.8569	0.7801	0.8078	0.7475	0.5675	0.6452	0.7301	0.7728	0.6927	0.6309	0.6604	0.6309	0.7792	0.5052	0.5303	0.5174	0.7772	0.5803
Radial SVM	0.8405	0.8619	0.8511	0.768	0.7989	0.7249	0.5503	0.6256	0.7152	0.7602	0.777	0.6459	0.7054	0.6479	0.8096	0.4998	0.5741	0.5343	0.7721	0.5741
Logistic Regression	0.8518	0.8632	0.8575	0.7815	0.8087	0.7401	0.5945	0.6594	0.7375	0.7763	0.7444	0.667	0.7036	0.667	0.8051	0.5073	0.5803	0.5413	0.7842	0.5803
k-NN	0.8122	0.8351	0.8235	0.7245	0.7613	0.6726	0.5503	0.6054	0.6985	0.7388	0.6922	0.6411	0.6657	0.6411	0.7792	0.4757	0.4919	0.4836	0.6778	0.4919
Decision Tree	0.8083	0.8029	0.8056	0.7057	0.7417	0.5394	0.5381	0.5387	0.6433	0.6645	0.6644	0.6688	0.6666	0.6615	0.7524	0.4387	0.4375	0.4381	0.6053	0.4509
XGBOOST	0.8533	0.8579	0.8556	0.7815	0.8069	0.7005	0.6093	0.6517	0.7301	0.7629	0.7266	0.6686	0.6964	0.6686	0.798	0.5408	0.575	0.5573	0.7563	0.575
Light GBM	0.8626	0.8672	0.8649	0.7955	0.8194	0.7122	0.6019	0.6524	0.7313	0.7665	0.7328	0.6747	0.7026	0.6747	0.7533	0.5699	0.583	0.5764	0.7712	0.583
PMbpd	0.9441	0.7466	0.8338	0.8952	0.8019	0.7008	0.6732	0.7754	0.824	0.7764	0.7082	0.7591	0.7793	0.8277	0.7668	0.5045	0.5776	0.5672	0.7825	0.5777
TS-PMbpd	0.952	0.7185	0.8189	0.8966	0.8199	0.6651	0.7076	0.7653	0.8253	0.7639	0.7039	0.7523	0.7764	0.823	0.7641	0.5223	0.5912	0.5534	0.7855	0.5912

BPD, bronchopulmonary dysplasia; BPD 0, no BPD; BPD 1, mild BPD; BPD 2, moderate BPD; BPD 3, severe BPD; SVM, support vector machine; k-NN, k-Nearest Neighbor; XGBOOST, Extreme Gradient Boost; GBM, Gradient Boost Machine.

### 3.3. Importance analysis by SHAP

**Figure 4:**
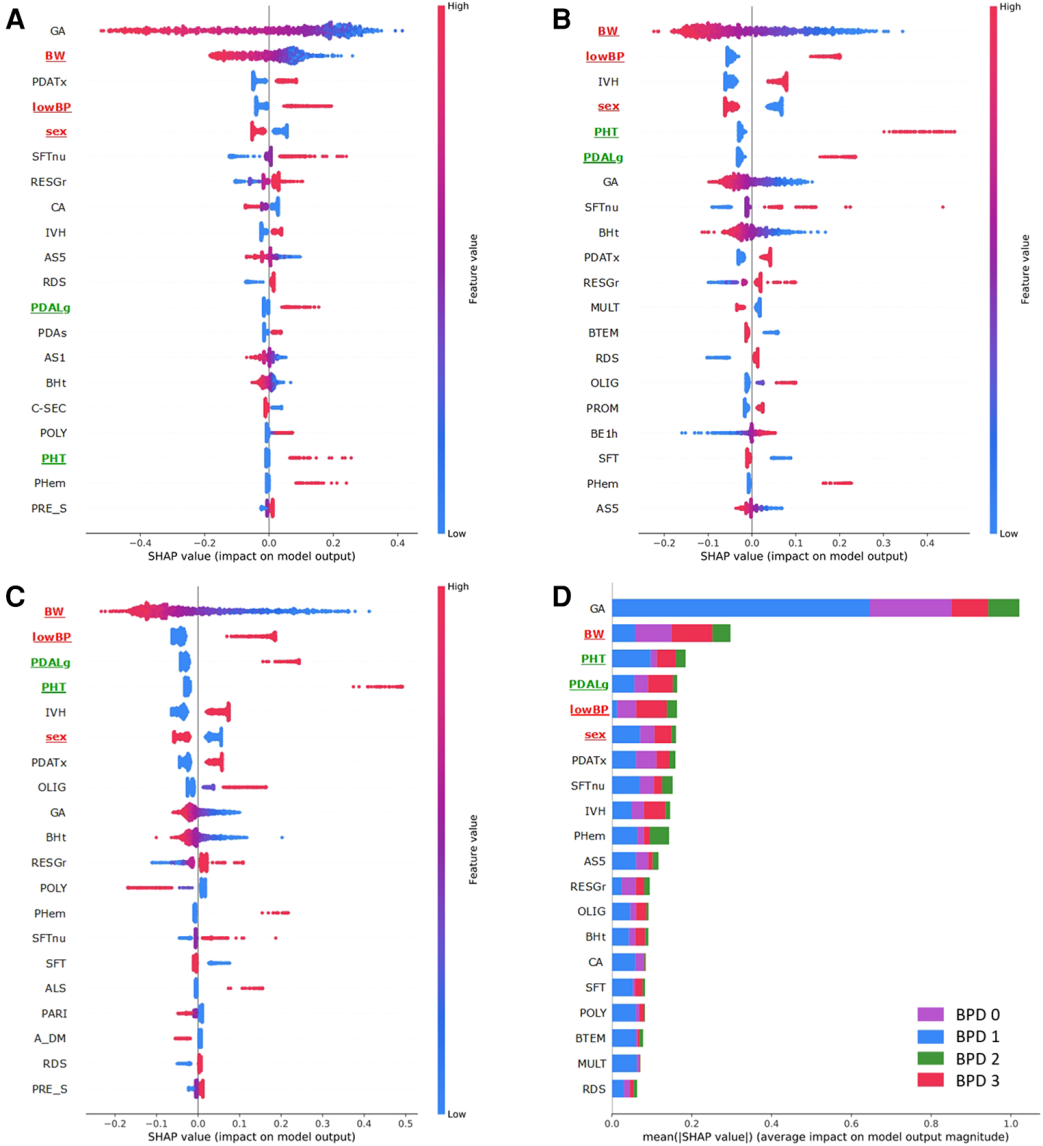
Top 20 variables contributions for BPD prediction by SHAP. (**A**) SHAP summary and importance matrix plots of the TS-PMbpd model in binary classification (BPD 0 vs. BPD 1,2,3). (**B**) SHAP summary and importance matrix plots of the TS-PMbpd model in binary classification (BPD 0,1 vs. BPD 2,3). (**C**) SHAP summary and importance matrix plot of the PMbpd model for binary classification (BPD 0,1,2 vs. BPD 3). (**D**) SHAP summary and importance matrix plot of the TS-PMbpd model in multi-classification (BPD 0 vs. BPD 1 vs. BPD 2 vs. BPD 3). In the dotted plot on the left, each dot represents one patient per feature, where red represents a higher value in continuous and ordinal variables (or positive correlations in categorical variables) and blue represents a lower value in continuous and ordinal variables (or negative correlations in categorical variables). The bar plot on the right presents the importance of each clinical variable in predicting the severity of BPD in VLBW infants in each model. The variable names in the top six for all classifications are highlighted in red, and top six in three out of four classifications are highlighted in green.

After producing ANN models for each binary and multi-classification, we sorted the top 20 variables that contributed the most to predicting the outcomes. Figure 4 depicts the importance matrix plot and the SHAP summary plot designated for the ANN models. The principal variables that contributed to the diagnosis of BPD in VLBW infants were GA, BW, PDA treatment, and low BP (Figure 4A). Among the principal variables for the presence of BPD ≥2 and 3 in VLBW infants, the first two variables in the top six were BW and low BP, in the same order. The latter four were IVH, sex, PHT, and PDA ligation, which had the same composition but different orders (Figures 4B,C). The principal variables that helped the diagnosis of each BPD severity in VLBW infants were GA, BW, PHT, PDA ligation, low BP, and sex (Figure 4D). In particular, BW, low BP, and sex were in the top six in all classifications, and PDA ligation and PHT were in the top six in three out of four classifications. In general, we found that low BP, male sex, PHT, PDA ligation, and PDA treatment were positively correlated with BPD severity, and GA and BW were negatively correlated.

## 4. Discussion

This national cohort study developed new ML models enhanced with a complementary MLP network (PMbpd) and an additional stepwise approach (TS-PMbpd) using antenatal and early postnatal clinical variables and compared their predictive power for the prediction of BPD and its severity using antenatal and early perinatal clinical variables. Our prediction model outperformed conventional logistic regression and other ML methods (SVM, k-NN, decision tree, XGBOOST, and Light GBM). We identified that GA, BW, and PDA treatment were significant variables for the occurrence of BPD and BW, and low BP for both BPD ≥2 and 3. Moreover, GA, BW, and PHT were the most important variables that predicted BPD severity in VLBW infants. Notably, BW, low BP, and sex were in the top six in all classifications, and PDA ligation and PHT were in the top six in three out of four classifications.

Although the SHAP value cannot be an absolute risk criterion, it is known to provide a common sense of the importance of each variable for individual predicted values (22). Therefore, it is possible to indirectly find out which variables affect the severity of BPD through the SHAP value. Antenatal Steroids, chorioamnionitis, fetal growth restriction, gestational age, birth weight, and sex are well-known prenatal risk factors for BPD. Postnatal factors include mechanical ventilation, postnatal steroids, patent ductus arteriosus, supplemental oxygen, and sepsis (24). In addition to well-known risk factors, in this study, low BP (25) and PHT (26), which required treatment within one week, were important as early factors for BPD. Previous studies (25, 26) have shown that the above variables play a role in the risk factors of BPD, but their importance is higher than expected, so attention should be paid to premature care and additional studies are needed in the future.

Several investigations have been intensively conducted to predict BPD and various severities in recent years, as poor short- and long-term outcomes have occurred in patients with BPD, especially in those with moderate and severe BPD (27). Most existing prediction tools have implemented traditional statistical methods for predicting BPD risk, mainly from smaller datasets or a single center, and have focused on the early prediction of BPD. Few have focused on its severity, although the three levels of severity have different outcomes (7, 27). Additionally, such models often have variable accuracy and yield inconsistent findings, leading to confusion or uncertainty among healthcare providers regarding the model to be used.

Recently, ML models have been promising prediction tools and have been used in numerous clinical applications, with the advantage of being able to minimize the error between predicted and observed outcomes. They applied ML algorithms such as logistic regression, XGBOOST, gradient-boosting decision trees, and random forests (27). Our study takes a step forward in identifying the clinical risk factors and developing effective early prediction models for BPD and its severity by securing a large number of BPD patients from a nationwide cohort registry and implementing a new deep learning technique. Although classic ML models are used for prediction, neural approaches have shown remarkable results in solving complex problems with big data. In addition, owing to their flexibility in designing the architecture of models and their nonlinearity, ANN often surpasses other ML methods in treating big data with complex distributions. Therefore, we first attempted to create a simple MLP model (PMbpd) that predicts BPD with factors that may occur prenatally and early after birth (usually within 1 week). Subsequently, we developed TS-PMbpd, which can provide various interpretations for input variables by creating the architecture of the model in two stages. Concatenating as our two-stage method is often used to improve performance in the computer vision field (28, 29) and is further used for classification problems (30) with insufficient data for securing diversity in feature interpretation, such as each severity prediction in the paper.

The KNN database is a nationwide cohort registry that includes approximately 75%–80% of VLBW infants born in South Korea and contains antenatal, postnatal, and long-term neurodevelopmental data. Our study sought to determine which factors were significant predictors of BPD in the NICU. Specifically, we selected the top 20 out of 45 variables to select the appropriate features. It is thought that it would be beneficial to reduce the variables a little more in future research to create compact modeling and go through the validation process. Additionally, by pooling VLBW infants from 77 different NICUs with a wide range of clinical conditions, neonatologists' preferences, and therapeutic protocols, we assume that our enhanced models could be feasible as BPD prediction tools in tertiary NICU settings that manage VLBW infants.

Respiratory support, especially for ventilator-induced lung injury, plays an important role as an independent risk factor in BPD development (13–15, 31). Therefore, in the first step of modeling, we chose variables to predict this factor and subsequently developed a two-stage model (TS-PMbpd) in a stepwise fashion to improve the predictive power. This could be confirmed by the improvement in performance for the BPD multi-classification problem, which is a problem with a small number of cases. This TS-PMbpd model showed its strength in predicting the severity and presence of BPD.

This study had a few limitations. BPD-related data, such as biomarkers, clinical symptoms, vital signs, and radiologic findings, could not be included because only data were collected from the KNN. Second, it was difficult to apply several clinical parameters to the model development because the exact timing of the occurrence was not recorded in a nationwide registry. Third, the longitudinal follow-up of variables during the NICU stay was not included in this model because each NICU has a different decision-making policy. Finally, it is difficult to determine the meaning of each parameter in the models and how ML methods generate results because of the nature of self-extracted data from large datasets.

Future developments with our BPD prediction models should reflect the changing BPD definition of these years and the great importance of predicting severe BPD patients, who have a worse prognosis and require more intensive care and follow-up. Moreover, we are considering developing a scoring system that is easy to use in clinical practice by inferring and assigning weights to the top compact variables.

## 5. Conclusions

Using a nationwide VLBW infant cohort, we developed new ML models incorporating crucial BPD indicators (RSd) into a two-step analysis and found significant clinical variables to predict early BPD and its severity with high predictive accuracy. In particular, our TS-PMbpd model showed the best performance in predicting the multi-classification of BPD for each severity; therefore, it can be used as an adjunctive predictive tool in clinical NICU practice for the early stratification of BPD.

## Data Availability

The datasets generated and/or analyzed during the current study are not publicly available due to the Korean Neonatal Network's (KNN) publication ethics policy. All information about patients is confidential; however, it is available from the corresponding author upon reasonable request. Requests to access the datasets should be directed to Hyun-Kyung Park, neopark@hanyang.ac.kr.

## References

[1] Praprotnik M Stucin Gantar I Lucovnik M Avcin T Krivec U. Respiratory morbidity, lung function and fitness assessment after bronchopulmonary dysplasia. J Perinatol. (2015) 35:1037–42. 10.1038/jp.2015.124 26468933

[2] Thebaud B Goss KN Laughon M Whitsett JA Abman SH Steinhorn RH Bronchopulmonary dysplasia. Nat Rev Dis Primers. (2019) 5:78. 10.1038/s41572-019-0127-7 31727986 PMC6986462

[3] Shim SY Yun JY Cho SJ Kim MH Park EA. The prediction of bronchopulmonary dysplasia in very low birth weight infants through clinical indicators within 1 hour of delivery. J Korean Med Sci. (2021) 36:e81. 10.3346/jkms.2021.36.e81 33754511 PMC7985290

[4] Higgins RD Jobe AH Koso-Thomas M Bancalari E Viscardi RM Hartert TV Bronchopulmonary dysplasia: executive summary of a workshop. J Pediatr. (2018) 197:300–8. 10.1016/j.jpeds.2018.01.043 29551318 PMC5970962

[5] Sriram S Schreiber MD Msall ME Kuban KCK Joseph RM O’Shea TM Cognitive development and quality of life associated with BPD in 10-year-olds born preterm. Pediatrics. (2018) 141:e20172719. 10.1542/peds.2017-2719 29773664 PMC6317639

[6] Jobe AH Bancalari E. Bronchopulmonary dysplasia. Am J Respir Crit Care Med. (2001) 163:1723–9. 10.1164/ajrccm.163.7.2011060 11401896

[7] Peng HB Zhan YL Chen Y Jin ZC Liu F Wang B Prediction models for bronchopulmonary dysplasia in preterm infants: a systematic review. Front Pediatr. (2022) 10:856159. 10.3389/fped.2022.856159 35633976 PMC9133667

[8] Kwok TC Batey N Luu KL Prayle A Sharkey D. Bronchopulmonary dysplasia prediction models: a systematic review and meta-analysis with validation. Pediatr Res. (2023). 10.1038/s41390-022-02451-8. [Epub ahead of print] PMC10356605 36624282

[9] Ding L Wang H Geng H Cui N Huang F Zhu X Prediction of bronchopulmonary dysplasia in preterm infants using postnatal risk factors. Front Pediatr. (2020) 8:349. 10.3389/fped.2020.00349 32676490 PMC7333538

[10] Giannini HM Ginestra JC Chivers C Draugelis M Hanish A Schweickert WD A machine learning algorithm to predict severe sepsis and septic shock: development, implementation, and impact on clinical practice. Crit Care Med. (2019) 47:1485–92. 10.1097/CCM.0000000000003891 31389839 PMC8635476

[11] Na JY Kim D Kwon AM Jeon JY Kim H Kim CR Artificial intelligence model comparison for risk factor analysis of patent ductus arteriosus in nationwide very low birth weight infants cohort. Sci Rep. (2021) 11:22353. 10.1038/s41598-021-01640-5 34785709 PMC8595677

[12] Son J Kim D Na JY Jung D Ahn JH Kim TH Development of artificial neural networks for early prediction of intestinal perforation in preterm infants. Sci Rep. (2022) 12:12112. 10.1038/s41598-022-16273-5 35840701 PMC9287325

[13] Stoll BJ Hansen NI Bell EF Shankaran S Laptook AR Walsh MC Neonatal outcomes of extremely preterm infants from the NICHD neonatal research network. Pediatrics. (2010) 126:443–56. 10.1542/peds.2009-2959 20732945 PMC2982806

[14] Keszler M Sant’Anna G. Mechanical ventilation and bronchopulmonary dysplasia. Clin Perinatol. (2015) 42:781–96. 10.1016/j.clp.2015.08.006 26593078

[15] Gibbs K Jensen EA Alexiou S Munson D Zhang H. Ventilation strategies in severe bronchopulmonary dysplasia. Neoreviews. (2020) 21:e226–37. 10.1542/neo.21-4-e226 32238485

[16] Papile LA Munsick-Bruno G Schaefer A. Relationship of cerebral intraventricular hemorrhage and early childhood neurologic handicaps. J Pediatr. (1983) 103:273–7. 10.1016/s0022-3476(83)80366-7 6875724

[17] Shin SH Shin SH Kim SH Kim YJ Cho H Kim EK The association of pregnancy-induced hypertension with bronchopulmonary dysplasia—a retrospective study based on the Korean neonatal network database. Sci Rep. (2020) 10:5600. 10.1038/s41598-020-62595-7 32221404 PMC7101434

[18] Kingma DP Ba J. Adam: A Method for Stochastic Optimization. CoRR, abs/1412.6980 (2014). 10.48550/arxiv.1412.6980

[19] Clevert D-A Unterthiner T Hochreiter S. Fast and accurate deep network learning by exponential linear units (elus). arXiv preprint arXiv:151107289. (2015). 10.48550/arXiv.1511.07289

[20] Srivastava N Hinton G Krizhevsky A Sutskever I Salakhutdinov R. Dropout: a simple way to prevent neural networks from overfitting. J Mach Learn Res. (2014) 15:1929–58.

[21] Ioffe S Szegedy C. Batch normalization: accelerating deep network training by reducing internal covariate shift. Proceedings of the 32nd international conference on international conference on machine learning (2015): 37:448–56

[22] Rozemberczki B Watson L Bayer P Yang H-T Kiss O Nilsson S The Shapley Value in Machine Learning. arXiv preprint arXiv:220205594. (2022). 10.48550/arXiv.2202.05594

[23] Lundberg SM Lee S-I. A unified approach to interpreting model predictions. Proceedings of the 31st international conference on neural information processing systems (NIPS’17). Curran Associates Inc., Red Hook, NY, USA, 30:4768–77. 10.48550/arXiv.1705.07874

[24] Trembath A Laughon MM. Predictors of bronchopulmonary dysplasia. Clin Perinatol. (2012) 39:585–601. 10.1016/j.clp.2012.06.014 22954271 PMC3443959

[25] Song YH Lee JA Choi BM Lim JW. Risk factors and prognosis in very low birth weight infants treated for hypotension during the first postnatal week from the Korean neonatal network. PLoS One. (2021) 16:e0258328. 10.1371/journal.pone.0258328 34648528 PMC8516276

[26] Kim HH Sung SI Yang MS Han YS Kim HS Ahn SY Early pulmonary hypertension is a risk factor for bronchopulmonary dysplasia-associated late pulmonary hypertension in extremely preterm infants. Sci Rep. (2021) 11:11206. 10.1038/s41598-021-90769-4 34045608 PMC8160152

[27] He W Zhang L Feng R Fang WH Cao Y Sun SQ Risk factors and machine learning prediction models for bronchopulmonary dysplasia severity in the Chinese population. World J Pediatr. (2023) 19:568–76. 10.1007/s12519-022-00635-0 36357648 PMC10198877

[28] Hariharan B Arbeláez P Girshick R Malik J, editors. Hypercolumns for object segmentation and fine-grained localization. Proceedings of the IEEE conference on computer vision and pattern recognition (2015).

[29] Lin G Shen C Van Den Hengel A Reid I, editors. Efficient piecewise training of deep structured models for semantic segmentation. Proceedings of the IEEE conference on computer vision and pattern recognition (2016).

[30] Rahimzadeh M Attar A. A modified deep convolutional neural network for detecting COVID-19 and pneumonia from chest x-ray images based on the concatenation of Xception and ResNet50V2. Inform Med Unlocked. (2020) 19:100360. 10.1016/j.imu.2020.100360 32501424 PMC7255267

[31] Gilfillan M Bhandari A Bhandari V. Diagnosis and management of bronchopulmonary dysplasia. Br Med J. (2021) 375:n1974. 10.1136/bmj.n1974 34670756

