# Supplementary material for: Two-stage learning-based prediction of bronchopulmonary dysplasia in very low birth weight infants: a nationwide cohort study
Source: Front Pediatr. 2023 Jun 13;11:1155921. doi: 10.3389/fped.2023.1155921 (PMC10294267; doi:10.3389/fped.2023.1155921)
Supplement: Supplementary file 1 [file Table1.docx]

Supplementary Material

Two-staged Learning-based Prediction of Bronchopulmonary Dysplasia in Very Low Birth Weight Infants: A Nationwide Cohort Study

Jae Kyoon Hwang^1^*, Dae Hyun Kim^2^*, Jae Yoon Na^1^*, Joonhyuk Son^3^, Yoon Ju Oh^2^, Donggoo Jung^2^, Chang-Ryul Kim^1^, Tae Hyun Kim^4†^ & Hyun-Kyung Park^1†^

^1^ Department of Pediatrics, Hanyang University College of Medicine, 222 Wangsimni-ro, Seongdong-gu, Seoul 04763, Korea

^2^ Department of Artificial Intelligence, Hanyang University, 222 Wangsimni‑ro, Seongdong‑gu, Seoul 04763, Korea.

^3^ Department of Pediatric Surgery, Hanyang University College of Medicine, 222 Wangsimni‑ro, Seongdong‑gu, Seoul 04763, Korea.

^4^ Department of Computer Science, Hanyang University, 222 Wangsimni‑ro, Seongdong‑gu, Seoul 04763, Korea

***** These authors contributed equally to this work.

†Correspondences: Hyun-Kyung Park,

[neopark@hanyang.ac.kr](mailto:neopark@hanyang.ac.kr); Tae Hyun Kim, [taehyunkim@hanyang.ac.kr](mailto:taehyunkim@hanyang.ac.kr)

# Supplementary Table S1. Clinical Variables and Abbreviations Used in the Analysis

| **Nominal variables (29 variables)** |
| --- |
| Oligohydramnios (OLIG); Polyhydramnios (POLY); Respiratory distress syndrome (RDS); Need for surfactant (SFT); Multiple gestation (MULT); Maternal overt diabetes mellitus (O_DM); Maternal all types of diabetes mellitus (A_DM); Maternal chronic hypertension (C_HTN); Maternal all types of hypertension (A_HTN); Histological chorioamnionitis (CA); Premature rupture of membranes (PROM); Delivery mode (C-SEC); Sex (sex); Need for initial resuscitation (RESU); Small for gestational age (SGA); *In vitro* fertilization (IVF); Congenital infection (C_INF); Body temperature <36 ℃ within 1 hour of birth (BTEM); Air leak syndrome (ALS); Massive pulmonary hemorrhage (PHem); Sepsis within 1 week of age (SEPS); Fungal infection within 1 week of age (FUNG); Meningitis within 1 week of age (MENI); Symptomatic patent ductus arteriosus (PDAs); Patent ductus arteriosus with any treatment (PDATx); Patent ductus arteriosus with surgical closure (PDALg); Patent ductus arteriosus with surgical closure within 1 week of age (PDALg7); Hypotension with medication within 1 week of age (lowBP); Pulmonary hypertension with treatment within 1 week of age (PHT) |
| **Ordinal variables (4 variables)** |
| Prenatal steroid use (PRE_S); Intraventricular hemorrhage (IVH); Number of administered surfactant (SFTnu); Degree of initial resuscitation (RESGr) |
| **Continuous variables (12 variables)** |
| Maternal age (M_AGE, years); Gravida (GRAV); Parity (PARI); Gestational age (GA); Apgar score at 1 min (AS1); Apgar score at 5 min (AS5); Birth weight (BW, gram); Birth weight z-score (BW_z); Hydrogen ion concentration in the blood within 1 hour after birth (pH1h); Base excess within 1 hour after birth (BE1h); Birth height (BHt, cm); Birth head circumference (BHC, cm) |

# Supplementary Table S2. All variables of the study participants

|  | BPD 0 (n=3,724) | BPD 1 (n=3,383) | BPD 2 (n=1,375) | BPD 3 (n=2,695) | *P*-value |
| --- | --- | --- | --- | --- | --- |
| SEX (male) | 2,006 (53.9) | 1,710 (50.5) | 632 (46.0) | 1,230 (45.6) | <0.001 |
| GA, weeks | 30.04 ± 1.17 | 27.9 ± 1.65 | 27.61 ± 1.99 | 27.00 ± 2.12 | <0.001 |
| AS1 | 5.57 ± 1.79 | 4.53 ± 1.87 | 4.06 ± 1.83 | 3.95 ± 1.81 | <0.001 |
| AS5 | 7.61 ± 1.37 | 6.8 ± 1.66 | 6.37 ± 1.76 | 6.29 ± 1.79 | <0.001 |
| BW, gram | 1,259.19 ± 183.51 | 1,064.39 ± 224.15 | 1,010.93 ± 245.57 | 885.79 ± 250.51 | <0.001 |
| BW_z | -0.01 ± 0.01 | 0.01 ± 0.54 | 0.06 ± 1.19 | 0.15 ± 1.84 | <0.001 |
| O_DM | 48 (1.3) | 48 (1.4) | 21 (1.5) | 44 (1.6) | 0.709 |
| A_DM | 449 (12.1) | 394 (11.6) | 153 (11.1) | 219 (8.1) | <0.001 |
| C_HTN | 92 (2.5) | 79 (2.3) | 36 (2.6) | 66 (2.4) | 0.95 |
| A_HTN | 899 (24.1) | 526 (15.5) | 241 (17.5) | 539 (20.0) | <0.001 |
| CA | 917 (24.6) | 1,139 (33.7) | 500 (36.4) | 997 (37.0) | 0.05 |
| pH1h | 7.27 ± 0.09 | 7.27 ± 0.1 | 7.26 ± 0.1 | 7.26 ± 0.11 | <0.001 |
| BE1h | -4.73 ± 3.16 | -5.15 ± 3.38 | -5.25 ± 3.47 | -5.51 ± 3.62 | <0.001 |
| RDS | 2,576 (69.2) | 3,121 (92.3) | 1,282 (93.2) | 2,565 (95.2) | <0.001 |
| SFT | 2,543 (68.3) | 3,161 (93.4) | 1,291 (93.9) | 2,575 (95.5) | <0.001 |
| SFTnu | 0.77 ± 0.61 | 1.20 ± 0.63 | 1.31 ± 0.79 | 1.38 ± 0.76 | <0.001 |
| PDATx | 735 (19.7) | 1,493 (44.1) | 686 (49.9) | 1,626 (60.3) | <0.001 |
| PDALg | 53 (1.4) | 325 (9.6) | 231 (16.8) | 778 (28.9) | <0.001 |
| PHT | 21 (0.6) | 78 (2.3) | 76 (5.5) | 453 (16.8) | <0.001 |
| lowBP | 195 (5.2) | 652 (19.3) | 412 (30.0) | 1,237 (45.9) | <0.001 |
| M_AGE | 33.23 ± 4.25 | 33.29 ± 4.31 | 33.22 ± 4.41 | 33.35 ± 4.15 | 0.683 |
| GRAV | 1.90 ± 1.17 | 2.02 ± 1.24 | 1.97 ± 1.21 | 1.93 ± 1.17 | <0.001 |
| PARI | 0.47 ± 0.73 | 0.52 ± 0.74 | 0.50 ± 0.71 | 0.45 ± 0.7 | 0.002 |
| OLIG | 401 (10.8) | 371 (11.0) | 159 (11.6) | 454 (16.8) | <0.001 |
| POLY | 33 (0.9) | 49 (1.4) | 12 (0.9) | 34 (1.3) | <0.001 |
| MULT | 1,470 (39.5) | 1,215 (35.9) | 431 (31.3) | 879 (32.6) | <0.001 |
| PROM | 1,390 (37.3) | 1,333 (39.4) | 585 (42.5) | 1,049 (38.9) | 0.008 |
| PRE_S | 2.36 ± 0.7 | 2.34 ± 0.7 | 2.34 ± 0.73 | 2.38 ± 0.7 | 0.074 |
| C-SEC | 2,994 (80.4) | 2,620 (77.4) | 1,036 (75.3) | 2,146 (79.6) | <0.001 |
| IVF | 962 (25.8) | 850 (25.1) | 338 (24.6) | 670 (24.9) | 0.744 |
| BTEM | 1.72 ± 0.69 | 1.65 ± 0.76 | 1.52 ± 0.86 | 1.51 ± 0.86 | <0.001 |
| SGA | 342 (9.2) | 167 (4.9) | 101 (7.3) | 445 (16.5) | <0.001 |
| BHt | 38.41 ± 2.38 | 36.29 ± 2.79 | 35.6 ± 3.19 | 34.27 ± 3.38 | <0.001 |
| BHC | 27.11 ± 1.49 | 25.54 ± 1.87 | 25.12 ± 2.12 | 24.41 ± 2.24 | <0.001 |
| RESU | 3,158 (84.8) | 3,245 (95.9) | 1,315 (95.6) | 2,625 (97.4) | <0.001 |
| RESGr | 2.89 ± 1.1 | 3.60 ± 0.89 | 3.7 ± 0.88 | 3.86 ± 0.81 | <0.001 |
| IVH | 1,006 (27.0) | 1,351 (39.9) | 725 (52.7) | 1,687 (62.6) | <0.001 |
| ALS | 42 (1.1) | 95 (2.8) | 68 (4.9) | 205 (7.6) | <0.001 |
| PHem | 15 (0.4) | 106 (3.1) | 64 (4.7) | 247 (9.2) | <0.001 |
| SEPS | 74 (2.0) | 124 (3.7) | 63 (4.6) | 142 (5.3) | <0.001 |
| FUNG | 1 (0.0) | 4 (0.1) | 3 (0.2) | 10 (0.4) | 0.007 |
| MENI | 1.0 ± 0.02 | 1.0 ± 0.02 | 1.0 ± 0.0 | 1.0 ± 0.02 | 0.922 |
| C_INF | 24 (0.6) | 34 (1.0) | 26 (1.9) | 47 (1.7) | <0.001 |
| PDAs | 555 (14.9) | 1,215 (35.9) | 602 (43.8) | 1,449 (53.8) | <0.001 |
| PDALg7 | 7 (0.2) | 35 (1.0) | 24 (1.7) | 59 (2.2) | <0.001 |

Values are expressed as numbers (%) or means (standard deviations). BPD, bronchopulmonary dysplasia; BPD 0, no BPD; BPD 1, mild BPD; BPD 2, moderate BPD; BPD 3, severe BPD; AS1, 1-minute Apgar score; AS5, 5-minute Apgar score; BW, birth weight; BW_z, birth weight z-score; O_DM, maternal overt diabetes mellitus; A_DM, maternal all types of diabetes mellitus; C_HTN, maternal chronic hypertension; A_HTN, maternal all types of hypertension; CA, histologic chorioamnionitis; pH1h, hydrogen ion concentration in the blood within 1 hour after birth; BE1h, Base excess within 1 h after birth; RDS, respiratory distress syndrome; SFT, need for surfactant; SFTnu, Number of administered surfactants; PDATx, patent ductus arteriosus with any treatment; PDALg, patent ductus arteriosus with surgical closure; PHT, pulmonary hypertension with treatment within 1 week of age; low BP, hypotension with medication within 1 week of age; M_AGE, maternal age; GRAV, gravida; PARI, parity; OLIG, oligohydramnios; POLY, polyhydramnios; MULT, multiple gestation; PROM, premature rupture of membrane; PRE_S, prenatal steroid; C-SEC, cesarean section; IVF, in-vitro fertilization; BTEM, body temperature <36 ℃ within 1 hour of birth; SGA, small for gestational age; BHt, birth height; BHC, birth head circumference; RESU, need for initial neonatal resuscitation at birth; RESGr, degree of initial neonatal resuscitation at birth; IVH, intraventricular hemorrhage; ALS, air leak syndrome; PHem, massive pulmonary hemorrhage; SEPS, bacterial sepsis within 1 week of age; FUNG, fungal sepsis within 1 week of age; MENI, meningitis within 1 week of age; C_INF, congenital infection; PDAs, symptomatic patent ductus arteriosus; PDALg7, patent ductus arteriosus ligation surgery within 7 days of age.
